# Optically tracked, single-coil, scanning magnetic induction tomography

**DOI:** 10.1117/1.JMI.4.2.023504

**Published:** 2017-06-16

**Authors:** Joe R. Feldkamp, Stephen Quirk

**Affiliations:** a Kimberly-Clark Corporation, Neenah, Wisconsin, United States; b Kimberly-Clark Corporation, Roswell, Georgia, United States

**Keywords:** electrical conductivity imaging, inductive loss, position tracking

## Abstract

Recent work has shown that single-coil, magnetic induction tomography (MIT) is useful for visualizing three-dimensional electrical conductivity distributions within biological targets. Coil-induced eddy currents and the associated secondary field are detected as an inductive loss while the coil is relocated to several unique positions and orientations near a target. Image reconstruction is then accomplished by inversion of a convolution integral that quantitatively maps inductive loss with conductivity. Previously, coil position and orientation had to be established by a template, which required assignment of fixed locations for the coil to visit. Here, our existing device is modified so that coil position and orientation are optically tracked while measuring inductive loss. Optical tracking is accomplished via a set of infrared reflective spheres mounted on the same enclosure that supports the coil. The coil center can be tracked with submillimeter accuracy while orientation angle is known to within a fraction of a degree. This work illustrates the use of single-coil MIT in full, position-orientation-tracked scan mode while imaging laboratory phantoms consisting of features having biologically relevant conductivity.

## 1 Introduction

Magnetic induction tomography (MIT) has been proposed in the last 25 years as a means to visualize the three-dimensional electrical conductivity of animal tissues.^1^^–^^6^ MIT has primarily been pursued via multicoil methods, while a recent advance has shown that single-coil MIT also provides a viable approach for conductivity imaging.^7^ In either case, radio frequency excitation is applied to a primary coil while in the vicinity of a conductive target, producing a field that creates eddy currents inside the target. The secondary field associated with the eddy currents can be detected by either measuring its effect on a second coil or by measuring impedance change in the primary coil itself.^8^^–^^11^ Though MIT may not match resolution performance shown in existing imaging modalities, it still offers promise as a portable, low-cost, modest-resolution tool able to image a property of the body not captured by other methods. It does so without contact and without the use of ionizing radiation or contrast agents. To the extent that disease states might exhibit abnormal conductivity, MIT could provide a tool to image disease onset or progression or response to treatment. As demonstrated in the work by Joines et al.,^12^ several tissues in the body exhibit a significantly elevated conductivity when malignant. Most notable from that work is the sevenfold conductivity increase in malignant breast tissue when compared to normal tissue. Also notable is the fourfold increase in relative permittivity for the same materials. In either case, measurements were made at room temperature on tissue specimens shortly after they were excised. Results with actual live tissue at body temperature may be different.^13^

We previously reported on our single-coil approach to MIT,^7^ which requires the relocation of a single coil, consisting of concentric circular loops lying within a common plane, to a number of locations in the vicinity of a target while measuring self-impedance change. Impedance change shows up as a dissipative resistive loss in series with the coil and is readily measured. Henceforth, impedance change is simply referred to as inductive loss. Inductive loss data, together with measured coil position and orientation, can be used for image reconstruction via a convolution integral that quantitatively links electrical conductivity and inductive loss. This mapping, which shows a linear dependence between inductive loss and conductivity distribution, has been validated numerous times against standard phantoms spanning a range of sizes.^7^^,^^14^^,^^15^ Though the mapping was derived under the assumption of uniform permittivity, linearity was found to be closely observed even in those instances when permittivity discontinuously changes to a greater extent than that found in biological specimens.^14^

In earlier work, position had to be tracked via a template that guided coil placement into a small number of predetermined positions and orientations. Though sufficiently accurate for preliminary evaluation, the template approach led to scan times of ∼30  min or longer and greatly limited sample size to fewer than ∼120 samples. Here, we remove that obstacle by optically tracking both coil position and orientation so that inductive loss can be sampled more efficiently. An optical body comprised of four infrared (IR) reflective spheres is mounted on the enclosure and then passively detected by a sensor that reports positions of individual spheres and body orientation, via quaternions, in the reference frame of the remote sensor. Using a pivot calibration step, a position vector from the optical body origin to the coil center can be determined. As a result, coil position can be determined to within ±0.3  mm and orientation to within a fraction of a degree.

In addition to testing that verifies position-tracking accuracy, the modified instrument is tested on laboratory phantoms to demonstrate correct synchronization of inductive loss measurements with position measurements. For example, inductive loss is measured while scanning over a relatively simple phantom, which is then directly compared to a theoretical prediction of loss over an identical virtual phantom. Given that localization of conductive features within a target is expected to be a common task for MIT, phantoms consisting of buried conductive features are scanned and processed by image reconstruction to determine whether free-style auto scanning MIT is able to discern if conductive features are near a phantom surface or located more deeply.

## 2 Optical Position and Orientation Tracking

**Fig. 1:**
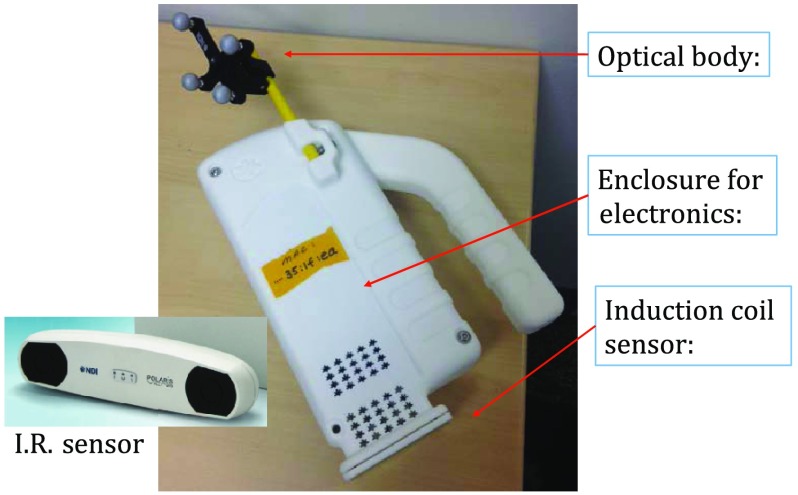
IR sensor and optical body mounted on attached mast.

The enclosure shown in Fig. 1 houses all electronics and provides support for mounting the printed circuit board (PCB)-type coil sensor. As shown, an insert was added for mast mounting an optical body from NDI (Northern Digital Inc., Canada).^16^^,^^17^ The optical body is one of the several standard configurations offered by NDI; it consists of four reflective spheres mounted in a common plane and is used to enable tracking of optical body orientation. The optical body is mounted on our instrument enclosure so that its reference plane is perpendicular to the coil plane. Data sent via RS-232 from the position and orientation tracking sensor provide the Cartesian coordinates of each sphere (one sphere is chosen as reference) and four quaternion values that specify optical body orientation.

**Fig. 2:**
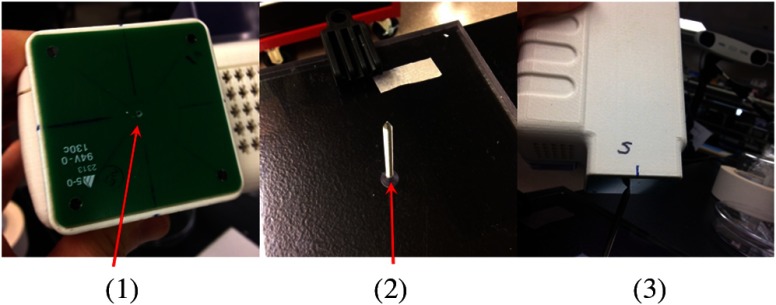
Steps used to enable relocation of the tracked position of the reference reflective sphere to the coil center. (1) Form divot at coil center. (2) Engage divot with pivot post. (3) Sweep enclosure in solid cone ∼30  deg.

**Fig. 3:**
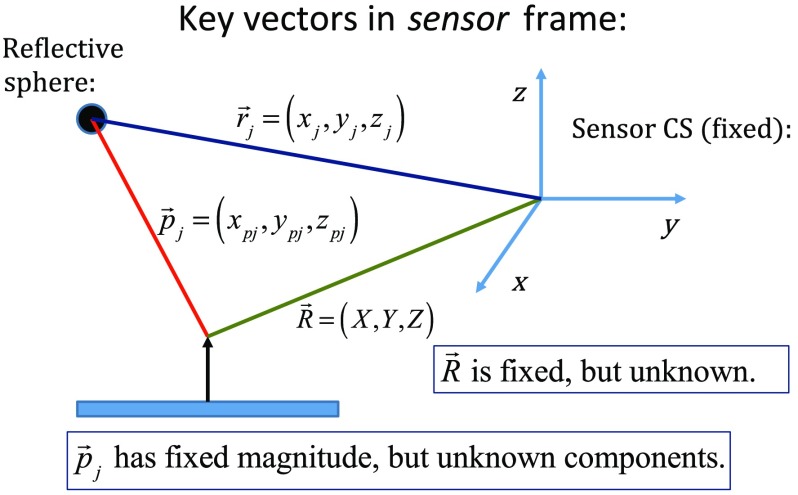
As shown, vector $\overset{\to }{R}$ is fixed, but unknown; vector ${\vec{p}}_{j}$ is variable but has fixed length; vector ${\overset{\to }{r}}_{j}$ is acquired from the sensor via RS-232 and locates the reference sphere. All vectors are in the frame of the sensor CS; CS, coordinate system.

Using an enclosure pivot procedure prior to scanning, the origin attached to the reference sphere can be relocated to the dead center of the induction coil. This is accomplished by introducing a divot at the coil center, engaging the divot on a fixed pivot post, and then sweeping the entire enclosure along a path that keeps the optical body within a solid cone of ∼30  deg. Position and orientation data are collected during the movement of coil and enclosure. Figure 2 shows images of the mechanical steps needed for calibration. Enclosure pivot data are processed by making use of the fact that the distance from pivot point to the reference sphere is fixed throughout the movement of the enclosure, as depicted in Fig. 3.

Also fixed is the coordinate system (CS) attached to the IR sensor supplied by NDI, so the position vector from the IR sensor to the pivot post is fixed, though unknown and to be determined. Relevant vectors are shown in Fig. 3. The fixed vector connecting the fixed pivot point and the fixed sensor is related to the other two vectors shown in Fig. 3 by

$$\overset{\to }{R}={\overset{\to }{r}}_{j}+{\overset{\to }{p}}_{j}.$$ (1)

The vector connecting the pivot post with the reflective [reference] sphere, though rotating during the pivoting step, has a fixed length. The fixed, unknown L2 norm for vector ${\overset{\to }{p}}_{j}$ may be directly computed as

$${\left\|{\overset{\to }{p}}_{j}\right\|}^{2}={\left\|\overset{\to }{R}-{\overset{\to }{r}}_{j}\right\|}^{2}={\left(X-x_{j}\right)}^{2}+{\left(Y-y_{j}\right)}^{2}+{\left(Z-z_{j}\right)}^{2};j=1,2,\ldots ,N.$$ (2)

Expanding and rewriting Eq. (2) leads to a linear problem in X, Y, and Z, provided that any one of the equations from (2) is used to eliminate the L2 norm of ${\overset{\to }{p}}_{j}$ from all the others, leaving

$$X(x_{i}-x_{j})+Y(y_{i}-y_{j})+Z(z_{i}-z_{j})=\frac{1}{2}(r_{i}^{2}-r_{j}^{2});j=1,2,3,\ldots ,N;\;\forall \;i<j.$$ (3)

N is the number of position samples collected during the pivoting procedure such that the full number of equations that may be written is equal to N*(N−1)/2; each equation is in turn used to eliminate the L2 norm of ${\vec{p}}_{j}$ from all other equations to generate all possible equations. A linear least squares type problem is then set up to determine the fixed length of vector ${\vec{p}}_{j}$ and the three coordinates associated with vector $\overset{\to }{R}$. Typically, ∼400 data points are acquired from the IR sensor during a conical sweep, so singular value decomposition (SVD) is used to find the components of $\vec{R}$. This pivot procedure is done only once prior to scans that simultaneously collect inductive loss and coil position-orientation data; the exception would be if the optical body is reattached.

Once the components of $\vec{R}$ have been found, Eq. (2) is used to find the magnitude of vector ${\vec{p}}_{j}$. Note that a value for $\left\|{\overset{\to }{p}}_{j}\right\|$ is found for each j; thus, an average is computed and used.

**Fig. 4:**
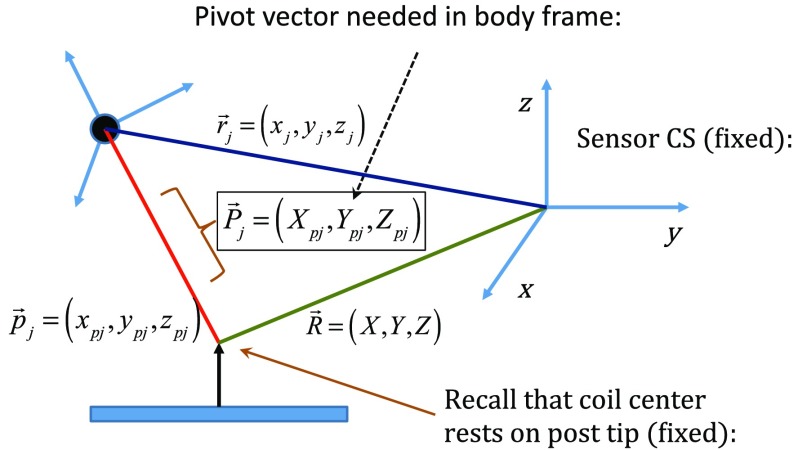
Illustration of relevant vectors—those in the frame of the fixed IR sensor and the single vector ${\vec{P}}_{j}$ in the frame of the optical body.

With $\vec{R}$ and $\left\|{\overset{\to }{p}}_{j}\right\|$ found, the vector ${\vec{P}}_{j}$ shown in Fig. 4, which is the pivot vector in the frame of the optical body, is needed. Since quaternions are also acquired at each one of the enclosure orientations, these may be used to build rotation matrices ${\overset{˜}{R}}_{j}$ that are then used to transform vector ${\vec{p}}_{j}$ from the sensor coordinate frame to the vector ${\vec{P}}_{j}$ in the body frame. Since there are ∼400 orientations, an average $\left\langle {\overset{\to }{P}}_{j}\right\rangle$ is found and subsequently used. Note that $\left\langle {\overset{\to }{P}}_{j}\right\rangle$ is fixed since the enclosure, together with the optical body, forms a rigid body. The key relations are given here

$$\overset{\to }{R}={\overset{\to }{r}}_{j}+{\overset{\to }{p}}_{j};\;\langle {\overset{\to }{P}}_{j}\rangle =\langle {\overset{˜}{R}}_{j}^{T}(\overset{\to }{R}-{\overset{\to }{r}}_{j})\rangle .$$ (4)

With vector $\left\langle {\overset{\to }{P}}_{j}\right\rangle$ known, any measurement of vector ${\vec{r}}_{j}$, together with optical body quaternions in our scanning MIT experiments, allows us to accurately locate the coil center

$${\overset{\to }{R}}_{\mathrm{ctr}}={\overset{\to }{r}}_{j}+{\overset{˜}{R}}_{j}\langle {\overset{\to }{P}}_{k}\rangle .$$ (5)

This is all done at the rate of 20 times per second. During single-coil MIT scans, the orientation of the coil is taken as the same as the orientation of the optical body since the Z-axis of the optical body and Z-axis of the coil are mechanically configured to be parallel.

## 3 Mesh Frame Coordinate System

**Fig. 5:**
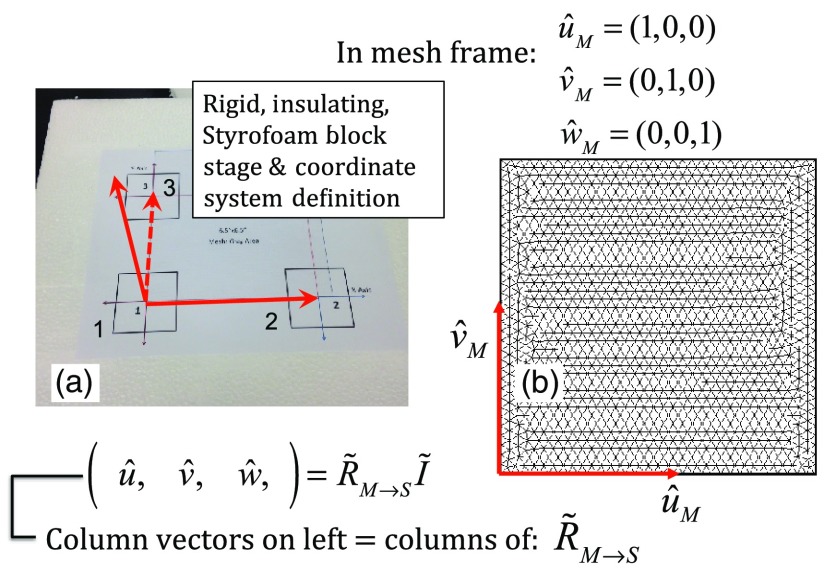
Mesh CS: (a) three position measurements define the mesh CS on the Styrofoam stage and (b) construction of unit vectors on Styrofoam stage.

Single-coil MIT scans are performed on samples contained within a 14-cm diameter Petri dish having a depth of ∼24  mm. The Petri dish is mounted on a ∼6-cm thick Styrofoam stage as shown in Fig. 5(a). For convenience, a second fixed reference frame is associated with the stage and defined by three measurements of coil position at select locations on the stage surface, as shown. Unit vectors shown in Fig. 5(b) define a mesh frame, which allows us to conveniently acquire scan locations within the finite-element mesh CS.

Three orthonormal vectors are easily computed from three position measurements, obtained by placing the square coil footprint sequentially into the three marked squares shown in Fig. 5(a)

u^=R→2−R→1|R→2−R→1|;v^=R→3−R→1|R→3−R→1|;w^=u^×v^. (6)

As written in Eq. (6), these three unit vectors are still represented in the reference frame of the IR sensor. These same three unit vectors in the mesh coordinate frame are just (1,0,0), (0,1,0), and (0,0,1). An orthogonal transformation ${\overset{˜}{R}}_{M\to S}$ connects these two sets of unit basis vectors

(u^,v^,w^)=R˜M→SI˜. (7)

Given that unit vectors attached to the Styrofoam stage are computed as in Eq. (6), Eq. (7) provides a straightforward approach for computing the rotation matrix needed to transform any vector in the sensor frame over to the mesh frame

$${\overset{\to }{R}}_{\text{Mesh}}={\overset{˜}{R}}_{M\to S}^{T}({\overset{\to }{R}}_{\text{Sensor}}-{\overset{\to }{R}}_{1}).$$ (8)

**Fig. 6:**
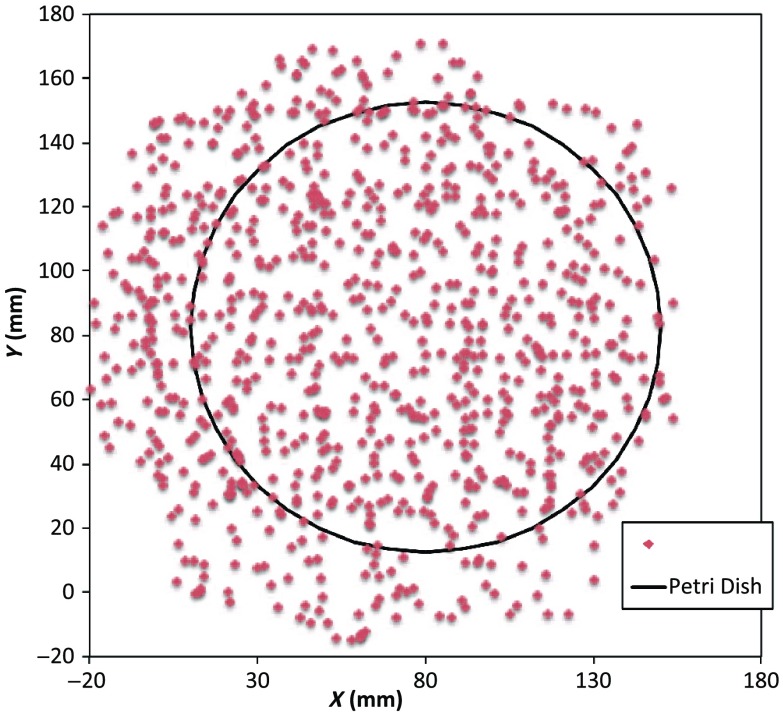
Scanning pattern illustrating how coil location is found in the CS of the mesh during a free-style scan.

Sensor frame vectors ${\vec{R}}_{1}$ and ${\overset{\to }{R}}_{\text{Sensor}}$ are obtained during the course of a scan so that after transformation via Eq. (8), we arrive at vector ${\vec{R}}_{\mathrm{Mesh}}$, which locates the coil in the frame of the mesh. As an illustration of coil localization in the mesh CS, Fig. 6 shows a set of (X,Y) coordinates obtained during the course of an auto scan over a Petri dish—that is, collecting position and coil loss data while moving the unit in a free-style manner across the stage. Clearly, there is a position sampling bias toward the left side of the dish. Since the enclosure is moved by hand, complete avoidance of sampling bias is not possible and is expected to have consequences during image reconstruction. Future work will consider oversampling, with subsequent removal of select samples to help restore sampling balance.

**Fig. 7:**
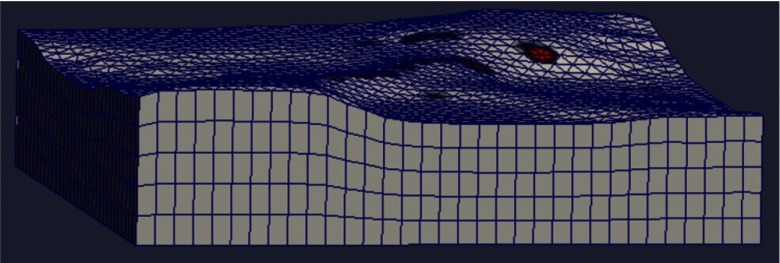
Illustration of mesh formation using position data acquired during a scan—the mesh is extruded to the height determined by the scan.

Scans can be done in either of two ways: one involves stepping the coil from one location to the next, while the second permits automatic acquisition of data while moving the coil enclosure in free form. In either case, scanning also permits us to establish the boundary of a specimen, which is useful for meshing purposes. The finite-element mesh used for image reconstruction is 16.5×16.5  cm and is extruded to the height of the specimen, as determined from the scan. Figure 7 shows an example that involves scanning over an irregular object to determine its upper boundary.

## 4 Inductive Loss Measurement

Coil geometry and construction have been described previously for several different designs.^7^^,^^18^ The coil used in this work has been described in detail in an older work^7^ and consists of five concentric circular loops on each of two planes, spaced 0.5 mm and prepared on a multilayer PCB. Loop radii are 4, 8, 12, 16, and 20 mm, while traces are 0.5-mm wide, built from 2 oz copper. All loops are wired in series, giving 10 total loops. There is a 1-mm buildup of PCB material on the side of the coil facing outward, giving at least a 1-mm separation between coil and target. Coil inductance L is calculated as described in Ref. 14, which was shown to be a reasonable approximation for our coil’s inductance provided that the distance between layers is very small compared with loop radii. Inductance for the coil used in this work was calculated to be 2.155  μH and shown to agree with experiment to within ±1%.^7^

Coil loss is computed from a change in the real part of admittance^14^ $\delta Y_{\mathrm{re}}$ relative to the free-space value, which subtracts the effect of any loss intrinsic to the coil. Given inductance L and frequency ω, coil loss is computed from the equation shown in Ref. 14, repeated here for convenience

$$\delta Z=\omega^{2}L^{2}\delta Y_{\mathrm{re}}.$$ (9)

Thus, two admittance measurements are needed: one in free space that avoids interaction with nearby conductive objects and subsequent measurements in the immediate vicinity of a conductive specimen. 2.0 Vpp fixed excitation is applied to the coil via a precision current-sensing resistor. Raw coil sensor voltages are first passed to a phase and gain detector (AD8302), with output from the AD8302 then sent to the controlling laptop via Bluetooth, which permits untethered operation. Current instrumentation measures admittance at 12.5 MHz with a precision of ∼±0.026  μS, which leads to a loss precision of ±0.00075Ω. However, other issues, most importantly drift, conspire to limit precision to ±0.0011Ω but represent an improvement over older instrumentation. Loss precision is measured by doing a “blank” scan over the Styrofoam stage. Additional details are found in Ref. 7—in particular, the approach for measuring phase angle difference between voltage and current in the induction coil.

## 5 Time Synchronization of Coil Loss and Position

Acquisition of coil loss and position-tracking data are handled through separate C++ libraries, though linked together via a common scanning application that manages both sensors. Each sensor has its own internal clock that keeps track of data acquisition times for either sensor. The position sensor clock advances at 20 Hz, while the coil sensor clock advances at a user selectable rate, which is set here at 5 Hz. Actual data collection is at the rate of 5 Hz in either case, so a data packet is acquired from the position sensor after every fourth clock pulse. Though each clock proceeds at a different rate, they both advance at constant rates, which is sufficient to enable synchronization of data. In fact, knowing the exact rate of clock advancement is not as important as knowing that clock rates are truly constant. Plotting the times associated with acquired position measurements against the times at which coil loss is measured yields a perfect straight line with an intercept that gives the offset time between the two clocks. The linear correlation is used to translate all measured coil loss times to the time they were acquired according to the position sensor clock. In this way, time of coil loss measurement is known according to the position sensor’s clock, to within a few microseconds. However, the two sets of events are still not synchronized; they are only recorded according to the same clock.

Before determining coil loss values at positions that correspond in time, raw admittance values are first passed through an 11-term Savitzky–Golay (SG) filter to suppress noise, which is known to improve signal-to-noise ratio. Future work plans to test discrete wavelet transform (DWT) denoising as it has been shown superior to SG.^19^ Subsequently, drift correction, which relies upon periodic interruption of the scan, is applied, allowing us to measure a free-space value of admittance—used to compute loss—so that a drift baseline can be determined and subtracted from smoothed admittance data. Baseline drift is presumed to be linear between successive free-space measurements of admittance.

**Fig. 8:**
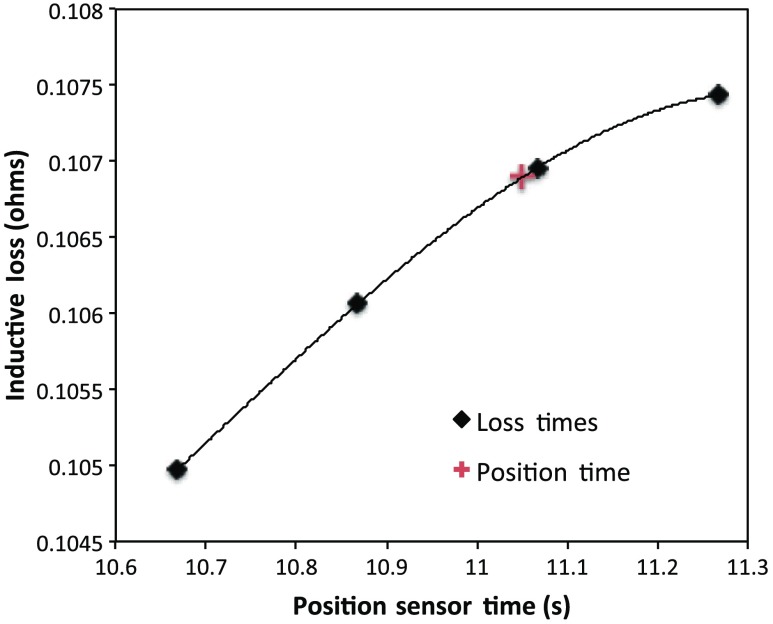
Cubic interpolation of four coil loss measurements to a time when position and orientation are measured.

To determine coil loss precisely at a time when the position was measured, the two sets of times are “lined up” to identify those position measurement times that are immediately preceded and followed by two inductive loss measurements. The four coil loss measurements that straddle the position measurement in this way are “exact-fitted” to a cubic polynomial, so a coil loss value can be computed at the exact time of a position measurement through interpolation. Figure 8 shows the interpolation process. Because calls to position and coil loss measurement routines occur very nearly at the same point in software, times of acquisition fall nearly on top of each other, reducing interpolation error.

## 6 Buried Phantom Construction

**Fig. 9:**
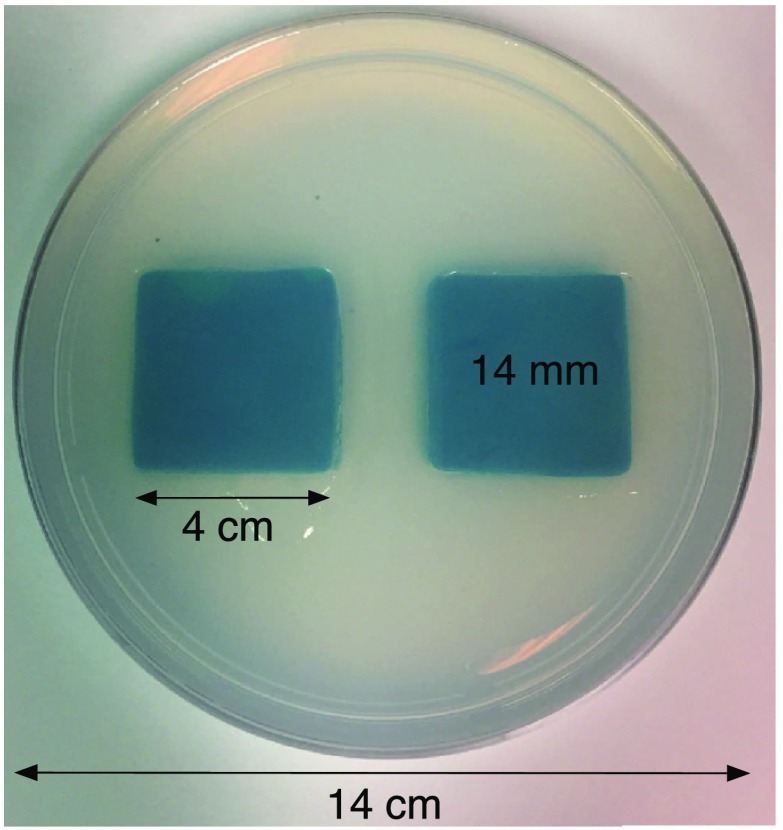
Phantom consisting of buried Play-Doh squares, immersed in agarose gel. Square inclusions are 4 cm on edge and buried to a depth of ∼14  mm beneath agarose.

The laboratory phantom used in this study is primarily designed to check the function of IR optical position tracking and its synchronization with inductive loss measurements. For that purpose, a relatively small phantom is used—certainly much smaller than the human body. Figure 9 shows the construction of a phantom consisting of two 40×40×8-mm thick squares of Play-Doh^™^ placed at the bottom of a 14×2.5  cm deep circular Petri dish and separated by ∼1.2  cm. Play-Doh^™^ conductivity was measured to be 4.35  S/m using a simple four-terminal sensing approach at 10 kHz, while material is contained within a 55×15  mm diameter plastic tube. The two Play-Doh^™^ features are immersed in agarose having a conductivity of 0.1  S/m, as described by Kandadai et al.,^20^ which we also measured using the four-terminal method at 10 kHz.

In addition to the task of instrumentation validation, another objective is to determine the ability of single-coil MIT to depth-resolve buried conductive features. Thus, the phantom is scanned twice: once with the Petri dish positioned so that conductive squares are at the bottom of the dish and a second scan while the Petri dish is upside down, leaving the conductive squares positioned at the top of the flipped dish from the point of view of the inductive sensor. During image reconstruction, key regularization parameters are set the same for both scan types, which avoids ambiguities. Furthermore, the scan is performed such that ∼600 samples are collected from each of three horizons above the Petri dish: first horizon on the Petri lid, a second at 2 mm above the lid, and a third at 4 mm above the lid. To get some sense for the importance of the third horizon of samples, image reconstruction is done with and without the third (upper) horizon of data.

## 7 Image Reconstruction Algorithm

Image reconstruction (inversion) is based on a mapping equation relating coil loss to coil position and orientation.^7^ Coil loss can be written as a convolution of conductivity $\sigma (\overset{\to }{r})$ and kernel $G({\vec{r}}_{c})$^14^^,^^21^

$$Z(\overset{\to }{c})=\int \sigma_{l}(\overset{\to }{r})G[{\overset{˜}{R}}^{T}(\overset{\to }{r}-\overset{\to }{c})]dxdydz.$$ (10)

The kernel is related to coil construction, as well as coil position and orientation in space

$$G({\overset{\to }{r}}_{c})=\frac{\mu^{2}\omega^{2}}{4\rho \pi^{2}}\underset{j,k}{\sum }\sqrt{\rho_{j}\rho_{k}}Q_{1/2}(\eta_{j})Q_{1/2}(\eta_{k}).$$ (11)

Arguments for the circularly symmetric toroid (or ring) function^22^ $Q_{1/2}$ lie in the interval 1<η<∞ and are related to field position by

$$\eta_{j}=\frac{\rho^{2}+\rho_{j}^{2}+z_{c}^{2}}{2\rho \rho_{j}}.$$ (12)

Using any suitable fixed laboratory CS, other symbols in Eqs. (10)–(12) are defined by

$\sigma (\overset{\to }{r})$, electrical conductivity (real part) at field position $\vec{r}=(x,y,z)$;
$\rho_{j}$, cylindrical radial distance from coil axis to wire loop “k”;
ρ, cylindrical radial distance from coil axis to field point;
$z_{c}$, perpendicular distance from coil plane to field point;
μ, magnetic permeability—considered uniform; and
ω, angular frequency.

Vector $\overset{\to }{c}$ connects the origin of the chosen fixed laboratory reference frame (usually origin of the mesh frame) with the coil’s center, while the vector ${\overset{\to }{r}}_{c}$ extends from the coil center to the field point, in the coil reference frame. Rotation matrix $\tilde{R}$ is available from the IR sensor at the rate of 20 Hz. After discretizing the convolution integral using deformed prismatic finite elements (∼20,000 elements), a system of equations is produced that predicts coil loss. A nonnegative least squares problem is set up and regularized via penalty matrix $\overset{˜}{D}$

$$\min\frac{1}{2}{\left\|\overset{˜}{A}\overset{\to }{\sigma }-\overset{\to }{Z}\right\|}_{2}^{2}+\frac{1}{2}\tau^{2}{\left\|\overset{˜}{D}(\overset{\to }{\sigma }-{\overset{\to }{\sigma }}_{\mathrm{avg}})\right\|}_{2}^{2}\;s.t.\;\overset{\to }{\sigma }\geq 0.$$ (13)

After converting problem Eq. (13) to standard form,^23^ image reconstruction proceeds via SVD of the matrix $\overset{˜}{A}{\overset{˜}{D}}^{-1}$. Solution nonnegativity is enforced through application of Karush–Kuhn–Tucker (KKT) multipliers and active set technology. The global regularization parameter is found by stepping τ through the singular values produced by the SVD, from largest to smallest. The process is stopped when the solution error norm approaches the inductive loss vector error norm from above—known as the discrepancy principle.^24^ Up to five iterations are needed for each singular value tested to satisfy KKT conditions. Because the kernel decreases with depth into a specimen (nearly exponential), the diagonal regularization matrix is set up to apply a smaller penalty as depth increases, mirroring the kernel itself. The solution of the minimization problem Eq. (13) is discussed at length in a recent publication^21^ and not covered here in detail. To facilitate image comparison, an identical black-body color scheme is used throughout: black (0  S/m); red (1  S/m); orange (2  S/m); yellow (3  S/m); and white (full scale).

## 8 Results

Clearly, implementation and coordination of all the data acquisition operations necessary for successful free-form, autoscanning single-coil MIT is no trivial task. Before getting too far ahead with the more interesting tasks of scanning and image reconstruction, it is essential that simpler experiments are done first to verify that the steps described thus far are sufficiently accurate. Thus, we focus initially on experiments that demonstrate accurate coil placement and synchronization with inductive loss measurements.

**Fig. 10:**
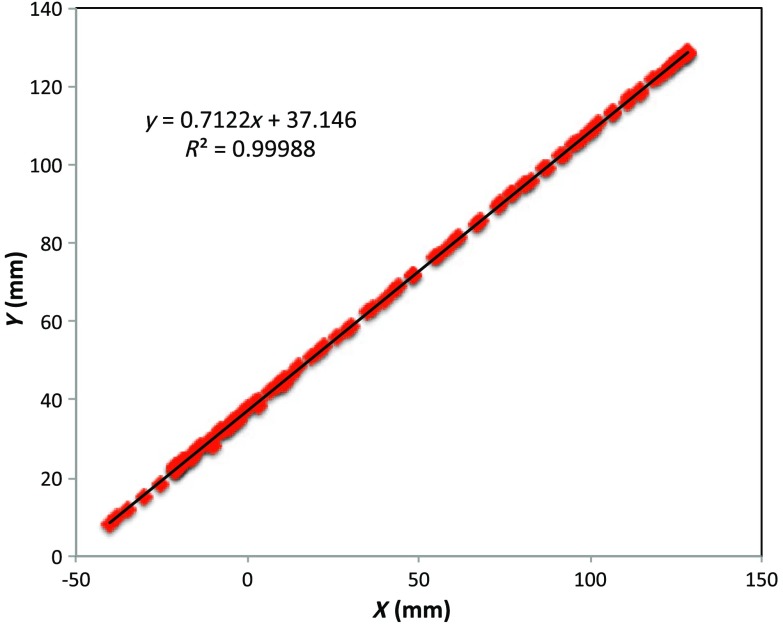
Result of a scan that tracks a nearly diagonal path along the upper surface of the Styrofoam stage, maintaining contact between coil and Styrofoam stage.

Figure 6 plotted measured XY-coordinates of the coil over the sample area in the mesh reference frame, which were obtained from a free-style test scan directly over the Styrofoam stage. This provides us with a basic test of tracking but gives no direct assurance of accuracy. If individual positions were known through separate trusted measurements, X and Y accuracy could be established. To that end, a precision ruler was taped approximately diagonally across the stage at a measured slope of 0.718 (mm/mm) and with one edge passing through the origin. Using the ruler’s edge as a guide, a scan consisting of 100 points was accomplished by sliding the enclosure along the straight edge, making sure to keep the coil PCB in reasonably good contact with the stage. A plot of the measured XY-coordinates is shown in Fig. 10, along with a linear least squares best fit.

The slope of the best line, 0.712 (mm/mm), is very close to that measured separately with a precision ruler (0.718). Furthermore, from the known coil dimensions (60×60  mm) and computed slope (0.712), the predicted intercept for the path of coil center is 36.831 mm. From Fig. 10 (fit equation shown in inset), the intercept error is 0.315 mm. Using the best fit straight line, a prediction of the Y-coordinate was subtracted from the sensor-measured Y-coordinate to provide an error estimate. The standard deviation of that error was computed as ±0.416  mm. Given that there is also some amount of operator error during the scan, such as modest flex of the straight edge, loss of full contact with the straight edge, or even imperfection in the straight edge itself, precision is expected to be better than ±0.416  mm—and suggested by the smaller intercept error.

Since an effort was made to keep the coil in contact with the stage, the error in the Z measurement can also be assessed. A precision straight edge was used to verify that the stage is indeed flat, to the extent that the straight edge is truly straight. Thus, for our purposes, the variation of the Z-coordinate can also be computed from the same data set. Scan data examined in this way were found to give a Z standard deviation of ±0.137  mm, well within the ±0.25  mm standard deviation claimed by NDI.^17^ An “equivalent” voxel size of ∼1.9  mm on edge can be computed for the finite-element mesh that we use here for image reconstruction by simply dividing the mesh volume by the number of elements (∼20,000). Given this is nearly 10× larger than position tracking precision, optical tracking accuracy is clearly acceptable in the present application.

To verify correct synchronization of the measured position with inductive loss, we performed a free-style “vertical scan,” consisting of the acquisition of inductive loss samples while the coil is gradually positioned farther away from a test conductive specimen. The specimen consisted of a 40×40×6-mm thick square of Play-Doh^™^ placed inside a 14×2.5  cm circular Petri dish, so the square was just beneath and in contact with the lid. Its conductivity was measured to be 4.35  S/m as described in Sec. 6. While scanning, the coil/enclosure is manually moved along an imaginary vertical line passing through the center of the square, with the coil maintained parallel to the Styrofoam stage—to the extent that operator technique allowed.

**Fig. 11:**
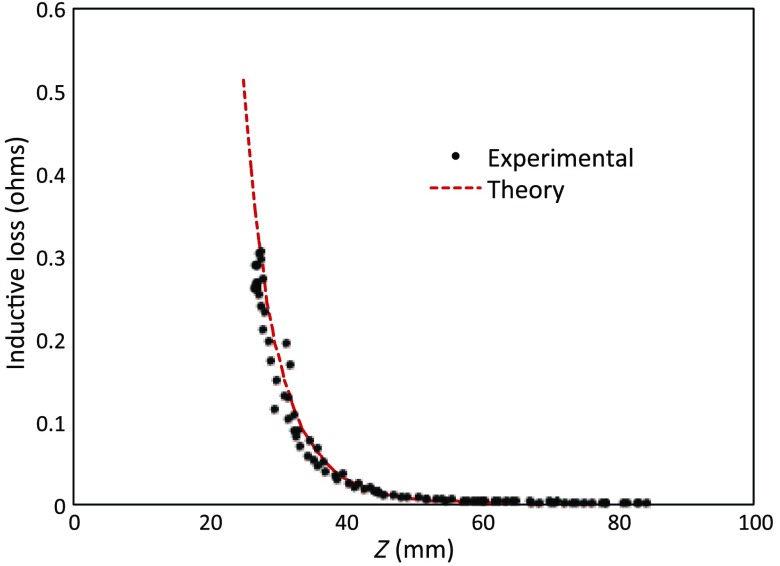
Inductive loss measured along a vertical line above the center of a 40×40×6  mm conductive square ∼4.35  S/m. Theoretical maintains perfect centering over the conductive square while the hand scan does not, contributing some variability.

Measured inductive loss values, plotted alongside a theoretical prediction from Eq. (10), are shown in Fig. 11; they reveal that the signal decays over a zone of ∼2  cm above the specimen, indicating the range of useful measurement for this phantom. Figure 11 not only verifies correct functioning of synchronization and position tracking technology but also validates yet again the predictive capability of the convolution mapping equation. The small variability observed in experimental data along the theoretical curve is not the result of noise, but rather the inability of the operator to move the coil exactly along an imaginary vertical axis passing through the square center without rotation; we note that such exact movement is not needed during an actual imaging scan since position and orientation are tracked. Quality of agreement between theory and experiment has been shown to be excellent through numerous validation exercises, suggesting that successful image reconstruction should not be hindered by the convolution integral nor the very small errors associated with position tracking and synchronization. Rather, inductive loss measurement noise remains the primary obstacle to obtaining desired image reconstruction results. Since scans are entirely free-form, obtaining an optimal spatial distribution of sampling locations may also be a potential issue, as was suggested by Fig. 6.

Rapid decay of inductive loss as a coil is moved farther from a specimen, as shown in Fig. 11, could be anticipated from the structure of the kernel function, which shows rapid (approximately exponential) decay with distance away from the coil. Because of this behavior, our image reconstruction algorithm currently uses “depth-dependent” regularization via the diagonal regularization matrix discussed in Sec. 7. Finite-element nodes located at greater depth beneath the boundary are penalized less, as a means to relieve the bias inherent to the kernel. Otherwise, image reconstruction at locations farthest from the coil become more vulnerable to noise and subject to increased localization error. Even with such measures, previous work with this algorithm^21^ suggests that noise levels in our current instrumentation are still too high by a factor of ∼3.

Nevertheless, we proceed with free-style scans of our two-feature phantom, designed to specifically test the ability of the complete system to depth-locate the square, conductive features. Collection of inductive loss data during the course of a scan is restricted to a span well within the “decay zone” above the phantom, as indicated in Fig. 11. As mentioned earlier, ∼600 samples were collected on each of three horizons, spaced 2 mm apart with EVA foam, for a total of nearly 1800. The center location of the mesh is (X,Y)=(82.5,82.5)  mm. Of interest is the extent to which autosampling locations are spread evenly over the span of the target. Scanning while phantom features are located at the bottom of the target, median X for each of the three horizons was found to be at 69.5, 71.3, and 71.2 mm, while median Y was found to be 81.2, 86.2, and 82.7 mm, ordered from nearest to farthest sampling horizon. Corresponding median values for coil coordinates while scanning the phantom with features near the top are 66.4, 67.4, and 70.3 mm for X and 80.9, 66.6, and 70.3 mm for Y.

Clearly, manual scanning across the target leads to an imbalance in sampling—i.e., the coil tends to visit locations “left of center” more frequently for either scan. This is currently an issue that needs to be resolved since it is not straightforward to perform a scan that ensures a particular distribution of sampling locations. In this particular case, there was a “left-bias.” Other attempts could just as easily produce a “right-bias.” However, image reconstruction should tell us the extent to which it matters.

**Fig. 12:**
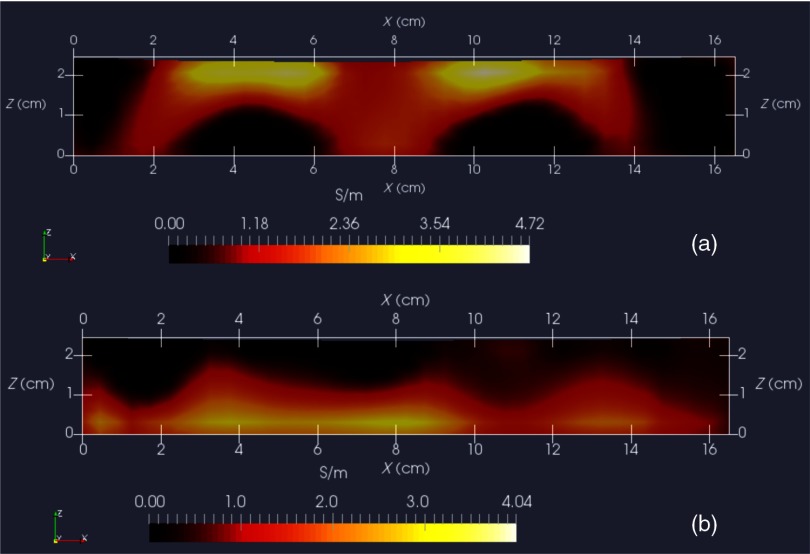
Y-normal slices through the phantom center: (a) conductive squares positioned at top and (b) conductive squares positioned at bottom.

For both phantom orientations, features at the bottom or top, Fig. 12 shows Y-normal slices cutting exactly through the phantom center. The images show that the rectangular features are indeed localized according to if they are near the bottom or near the top of the target. Furthermore, two distinct features are resolved when in the upper portion of the phantom, even though they are spaced only 1.2 cm apart. At the bottom, however, there is a loss of distinctiveness, which we attribute mostly to noise. Additional work is needed to determine if alternate sampling schemes will help with resolution. In both cases, it is clear that the left feature appears with greater clarity, which we attribute to skewed sampling.

**Fig. 13:**
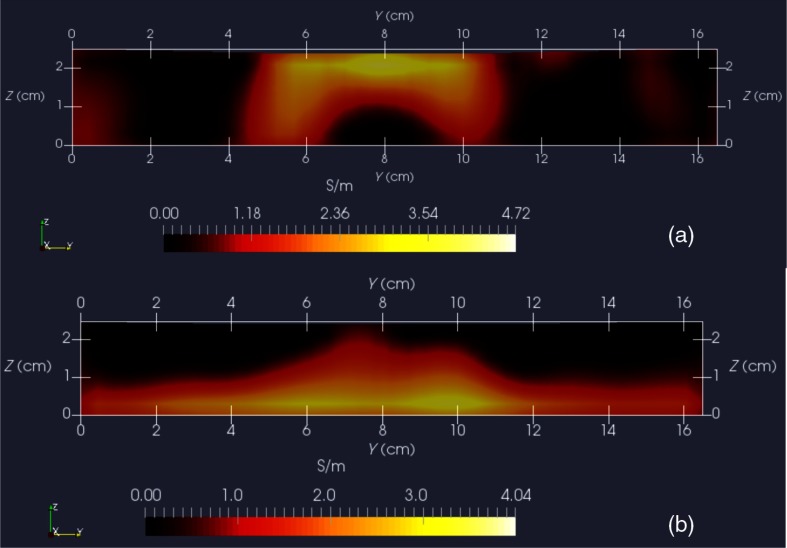
X-normal slices passing through center of left conductive feature: (a) conductive squares positioned at top and (b) conductive squares positioned at bottom.

Figure 13 shows X-normal image slices that pass through the left rectangular feature, whether at the bottom or top. Again, the images show that the immersed conductive feature correctly appears either near the top or bottom of the phantom, as appropriate. Especially in the case of bottom feature placement, there is considerable smearing of the conductive feature, which we attribute primarily to inadequate S/N performance—we noted earlier that this needs to be improved ∼3×.

**Fig. 14:**
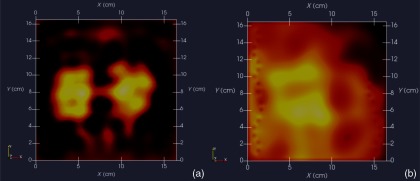
Z-normal slices: (a) slice obtained at Z=20  mm (or 4-mm depth) with conductive squares at top and (b) slice taken at Z=4  mm (or 20-mm depth) with conductive squares at bottom. Refer to Figs. 12 or 13 for corresponding scales.

Finally, Fig. 14 shows Z-normal slices that pass through the phantom at depths appropriate to either feature’s depth—slicing through the midpoint of the rectangular solid feature in either case. The two features clearly emerge when slicing near the top of the phantom, though the left feature more nearly adopts correct geometry. With both features at the bottom, however, only the left feature clearly appears, though enlarged. Here, both the effects of inadequate S/N and imbalance in sampling conspire to cause a disproportionate emphasis on the left feature. Throughout the bottom image slice, considerable smearing exists. This was expected, given the results of Feldkamp,^21^ which demonstrate that as noise is added to virtual scan data, increased blurring occurs that becomes even more pronounced at increased depths.

## 9 Discussion and Next Steps

As shown, IR optical sensing provides an effective tool for accurately tracking both position and orientation of a coil sensor used for single-coil MIT imaging. A data acquisition rate of 5 Hz was used for all scans in this study, so a full scan was completed in ∼6  min. In contrast, the previous template method of position tracking required ∼30  min just to acquire 132 samples. Crucial for practical tracking was the synchronization of coil position measurements with acquisition of inductive loss values. In our implementation, this was kept to within a few microseconds by coordinating clocks on both devices. In addition to verifying that the position-sampling accuracy is very close to that reported by Wiles et al.,^17^ further examples of free-form single-coil scanning are provided in the proceedings predecessor to this report.^16^ In particular, that work showcased single-coil MIT imaging of a 3-cm thick slab of well-marbled, cross-cut veal shank, indicating that fat, bone, and muscle can be distinguished.

A particular issue that arises with free-form scanning is the potential for sampling imbalance. Though this was not an issue when using templates that mirrored the strategy of Latin hypercube sampling, imbalance can cause a system that is inherently symmetrical, such as that used here, to appear asymmetrical under image reconstruction. Thus, a near term goal is to adjust the sampling software to automatically provide guidance on the extent to which balanced sampling is maintained during a scan. An alternative approach is to selectively remove data so that remaining data have a more desirable balance. Given that large data sets are now more easily obtained, a useful approach is to greatly oversample during scanning, so an adequately sized and “balanced” sample set remains after data pruning.

A related issue centers on the extent to which samples should be acquired at locations more distant from a target boundary. For larger targets, the “decay zone” over which the signal rolls off becomes even more extended, so some automated approach is needed to guide a scan to include an appropriate number of samples at locations more distant from the target. If too many samples are acquired near the target, depth discrimination may deteriorate. In fact, depth resolution was somewhat impaired in images shown in Figs. 12–14 when data acquired on the third horizon were removed—though XY fidelity remained intact. This suggests that the addition of yet another, fourth, horizon of data might have improved depth resolution still further. However, inclusion of more distant sampling starts to push the limits of instrumental S/N performance and could significantly corrupt image reconstruction, especially for the small phantoms scanned here. To help with sampling, but without overly burdening the operator, we plan to modify current software to provide a simple indication that sampling has become too biased or too distant from the target. Of course, S/N improvement can help with the latter issue, which we are currently addressing with more sophisticated denoising schemes, such as the DWT.^19^
